# A straightforward LC approach using an amine column and single quad mass detector to determine choline chloride in feed additives and feeds

**DOI:** 10.1016/j.mex.2017.09.001

**Published:** 2017-09-18

**Authors:** Fabio Granados-Chinchilla, Graciela Artavia

**Affiliations:** a Centro de Investigación en Nutrición Animal (CINA), Universidad de Costa Rica, Ciudad Universitaria Rodrigo Facio, 11501-2060 San José, Costa Rica; b Centro Nacional de Ciencia y Tecnología de Alimentos (CITA), Universidad de Costa Rica, Ciudad Universitaria Rodrigo Facio, 11501-2060 San José, Costa Rica

**Keywords:** Choline chloride in feeds, Choline chloride, LC/MS, Amine column, Feed, Feed additives

## Abstract

Considering choline (ChCl) as an essential ingredient for animals and that it is administered through feed, we developed an easy, accurate, and sensitive method for its analysis. The method is straightforward, derivatization-free and has no secondary chromatographic interactions.

• We demonstrated that the method can be used for quality control for feeds and feed additives containing choline chloride
• We report a simple chromatographic method which takes advantage of the hydroxyl moiety present in ChCl and a MS detector.
• We demonstrated that a single quadrupole detector is an effective option for the quantification of ChCl in feeds as an alternative for the more expensive tandem MS system.

## Graphical abstract

**Figure:**
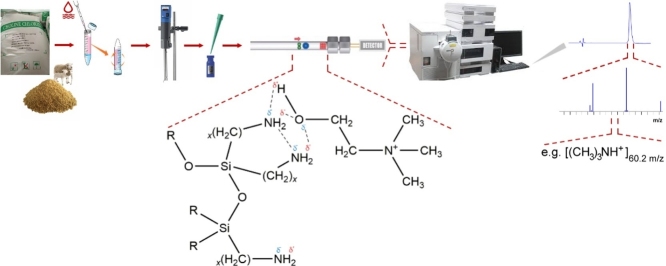

## Method details

### Background

Choline (2-hydroxy-*N*,*N*,*N*-trimethylethanaminium) is an essential nutrient belonging to the family of water soluble vitamin analogs, being the precursor for the neurotransmitter acetylcholine [1]. ChCl is a rather complicated analyte regarding detection as it lacks distinctive chromophores. Then, the use of traditional detection systems is limited or requires further derivatization. The most common approaches to assessing choline are by using ion chromatography, which uses specialized reagents to pair the choline ion or spectrophotometric analysis, which requires a colorimetric reagent and is time-consuming. However, more recent methods have been proposed using techniques such as nuclear magnetic resonance, mass spectrometry (MS), gas chromatography, capillary electrophoresis, UV–vis, thin layer chromatography [2], but limited mainly, to foods for human consumption. Though no maximum levels of choline chloride in feeds have been established in the EU; Council Directive 70/524/EEC8 approves its use in all species as a nutritional additive [3]. Although choline chloride has an ample application in feed formulations, few methods have been designed to test for this analyte [4], [5]. For example, AOAC^®^ has available several Official Methods^SM^ for ChCl determination on infant formula but none destined for the analysis of animal feed or feed ingredients. The aforementioned methods include: 999.14 (enzymatic colorimetric method), 2012.18 (UHPLC–MS/MS), 2012.20 [6] (ion chromatography and suppressed conductivity), 2014.04 (HILIC LC–MS/MS) and 2015.10 [7] (LC/MS/MS). Notwithstanding, in feed, choline is usually added in free form as a chloride salt. As with every feed ingredient, maximum or minimum limits must be declared on the feed label and said concentrations should be verifiable by analytical means [8]. Hence, an accurate method specifically tailored for feeds and, if possible, with few steps is paramount. Herein we report a simple chromatographic method availed of the ChCl hydroxyl group and a MS detector. Furthermore, we demonstrated that a single quadrupole detector is an effective alternative for the quantification of ChCl.

### Reagents

Acetonitrile (ACN, chromatographic grade), hydrochloric acid (36.5–38.0%, BAKER ANALYZED^®^ ACS Reagent) were purchased from J.T. Baker (Avantor Materials, PA, USA). ChCl was purchased from Sigma-Aldrich (≥99%, 239941, St. Louis, MO, USA).

### Sample extraction

A representative (1.0 ± 0.1) g subsample was used for testing, 5 mL of an 80 °C preheated 6 mol L^−1^ aqueous hydrochloric acid solution (to favor matrix components ionization, increase water polarity and extraction capacity) was added to the sample. The mixture was forced into contact and homogenized using a digital Ultra-turrax^®^ at 18 000 rpm (T25, IKA^®^ Werke GmbH & Co. KG, Staufen in Breisgau, Germany) during 1–3 min. The resultant mixture was centrifuged (at 2000*g* for 10 min). The supernatant recovered and filtered by pressure through a 0.45 and 0.22 μm (used sequentially, Acrodisc^®^ syringe filters with PVDF hydrophilic membrane, Pall Corporation, Port Washington, NY, USA) and recovered in an HPLC 2 mL vial for injection (Agilent Technologies, Santa Clara, CA, USA).

### Chromatographic conditions

All assays performed using an Agilent Technologies LC/MS system equipped with 1260 infinity quaternary pump (61311C), column compartment (G1316A), an automatic liquid sampler modules (ALS, G7129A) and a 6120-single quadrupole mass spectrometer with electrospray ionization ion source (Agilent Technologies, Santa Clara, CA, USA).

The isocratic analysis was performed at 0.20 mL min^−1^ using 20% acetonitrile and 80% water [type I, 0.055 μS cm^−1^ at 25 °C, 5 μg L^−1^ TOC obtained using an A10 Milli-Q Advantage system and an Elix 35 (Merck KGaA, Darmstadt Germany)]. A volume of 0.10 μL was injected into the system for additives and 1 μL for compound feed. We obtained a complete chromatographic run for ChCl under 10 min.

Considering the presence of an hydroxyl moiety within the choline structure, an amine based chromatographic column was used for analytical separation (Zorbax Carbohydrate Analysis, 4.6 mm ID × 150 mm, 5 μm, Agilent Technologies, Santa Clara, CA, USA). As the hydroxyl is a none ionizable functional group, it is more reliable for purposes of quantifying than the choline molecule’s aminium counterpart is.

### MS detection system conditions

**Fig. 1:**
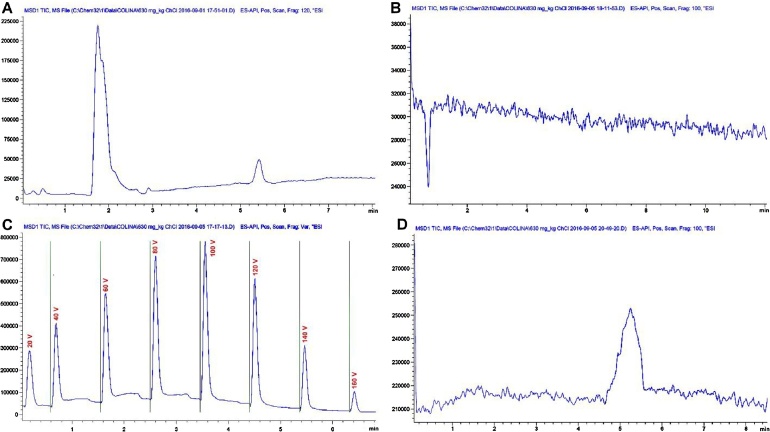
A. Total ion chromatogram (TIC, scan mode) of a 10 mg L^−1^ choline chloride_(*tr*_ _=_ _5.21)_ standard in aqueous solution. B. Signal to noise ratio experiment in TIC of a blank sample. C. Fragmentor voltage cycling from 20 to 160 V using flow injection analysis. D. Extinction experiment in TIC mode to determine sensitivity, 0.63 mg L^−1^ choline_(*tr*_ _=_ _5.11)_ standard solution.

Total ion chromatographs allows us to obtain the MS spectra for choline (Fig. 1A) and demonstrate no other substances coelutes during chromatography (Fig. 1B); though in MS analysis, coeluents are less problematic. Fragmenter was cycled to assess the voltage (from 20 to 160 V) that renders the highest sensitivity for the compound; omitting column interaction (Fig. 1C). Drying gas, nebulizer pressure, drying gas temperature and capillary voltage was set, respectively, to 12.0 L min^−1^, 50 psi, 350 °C, 4 000 V for positive ion mode electrospray ionization (ESI^+^). Still, the total ion chromatograph (scan mode, mass range, fragmentor and detector gain set to 90–200 *m*/*z*, 100 V and 1.00, respectively) is capable of analyzing 0.63 mg L^−1^ without issues (Fig. 1D). As expected, in selected ion monitoring (SIM mode, target ion, peak width and cycle time set to 104.10 *m*/*z*, 0.1 min, and 0.60 s cycle^−1^) sensibility augment considerably (i.e. 2.1 × 10^−2^ mg L^−1^).

We demonstrated that a single quadrupole gives a high sensitivity and accuracy determining ChCl with only a fraction of the cost (ca. 120 000 USD) when compared to a tandem MS system, which allows their installment in laboratories with relative ease. An inherent advantage of using a MS detector for the analysis of choline, is the fact that no ionic mobile-phase modifier is needed to assess this analyte.

### Method performance and validation parameters

Standard calibration curves were prepared using concentrations ranging from 0.63 to 5.42 mg L^−1^. The resulting general equation derived from five different calibrations curves was: *y* = (4.0 × 10^3^ ± 4.3 × 10^2^)*x +* (4.5 × 10^3^ ± 7.1 × 10^2^), with coefficients of determination (*r^2^*) ranging from 0.991 to 0.995, which demonstrates good linearity among the concentrations tested. The slope and intercept variability demonstrates run to run reproducibility.

Method sensibility was calculated using 3.3 times the signal-to-noise (S/N) ratio of a blank feed sample chromatogram and reassessed and verified by extinction assay using an extract from a feed spiked at a 1 mg kg^−1^ level. Feed was used instead of a feed additive as a blank, as we expect matrix effects from the more complex sample. Limits of detection and quantification (10 × S/N) were calculated at 20.3 and 62.0 μg L^−1^, respectively. The previous values result in 101.6 μg kg^−1^ and 310.0 μg kg^−1^ when expressed in the matrix. This sensibility is sufficient and exceeds requirements, for the purpose intended, as the common concentrations found in compound feeds are 1 × 10^4^ higher than the limit of detection.

Intraday and interday repeatability were estimated to be 7.4 and 10.4% RSD and 4.6 and 5.5% RSD at a 1 mg kg^−1^ and 1 000 mg kg^−1^spiking level, respectively. On both cases, calculations were performed using 6 independent replicates (Table 1).

**Table 1:** Precision and accuracy determinations of spiked samples with ChCl.

Replicate	1	2	3	4	5	6	Mean ± SD	%RSD
	Concentration, mg kg^−1^ (Recovery, %)							
Spike level, 1 mg kg^−1^								
Intraday	1.03 (91.5)	0.97 (86.2)	0.91 (80.9)	1.12 (99.5)	1.11 (98.7)	0.98 (87.1)	1.02 ± 0.08 (90.7 ± 6.7)	7.4
Interday	1.11 (98.7)	0.95 (84.4)	0.98 (87.1)	1.21 (107.5)	1.26 (112.0)	1.19 (105.8)	1.12 ± 0.12 (99.3 ± 10.3)	10.4
Spike level, 1000 mg kg^−1^								
Intraday	998.1 (100.1)	1 000.3 (100.3)	1 122.1 (112.5)	995.6 (99.8)	990.7 (99.3)	1 000.9 (100.4)	1 018.0 ± 46.7 (102.1 ± 4.7)	4.6
Interday	1 011.12 (101.3)	1 109.1 (111.1)	1 134.5 (113.6)	1 000.8 (100.4)	998.7 (100.0)	996.1 (99.8)	1 041.9 ± 57.2 (104.4 ± 5.7)	5.5

SD: standard deviation.% RSD: percentage relative standard deviation. Spike samples were prepared adding the necessary solid standard to a blank sample, real concentrations of 1.12 and 997.25 mg kg^−1^ calculated for the nominal spike levels of 1 and 1000 mg kg^−1^, respectively.

Theoretical plates (*N*), theoretical plates per meter, peak asymmetry factor (*A_s_*) and tailing factor (*T_f_*) were calculated to be 3 759, 25 063, 0.937 and 0.969, respectively. Data extracted from an average from *n* = 10 injections of the 0.63 mg L^−1^ standard in conditions of reproducibility. Altogether these values imply an adequate column efficiency and symmetric signals or peaks during elution (i.e., with no significant tailing or fronting). Theoretic plates were calculated using the half peak height method.

AAFCO check program sample code 201721, dry dog food, was used to assess accuracy. Though no *z* value was obtained for the sample, reported values ranged from 1 992.5 to 2 794.0 mg kg^−1^. We achieved for this case a concentration of (2 230.2 ± 244.7) mg kg^−1^ resulting in a 90.0–99.3 recovery. A result which is in line with the recoveries attained from the spiked samples at a 1 mg kg^−1^ (90.9–99.3%) and 1 000 mg kg^−1^ (102.1-104.4%) enrichment level (Table 1). As expected, lower concentrations assayed generate higher variation. AAFCO samples 201722 (Poultry layer feed) and 201724 (Beef feed, medicated) were also assessed obtaining values of (1 435.7 ± 157.5) mg kg^−1^ [round results reported 570–1 635 mg kg^−1^] and (1 011.0 ± 110.9) mg kg^−1^ [round results reported 993–1 600 mg kg^−1^]. The uncertainty of measurements was based on repeatability analysis and reported at a *k* = 2 level with a 95% confidence level (10.97% relative to ChCl concentration).

### Real sample application

**Fig. 2:**
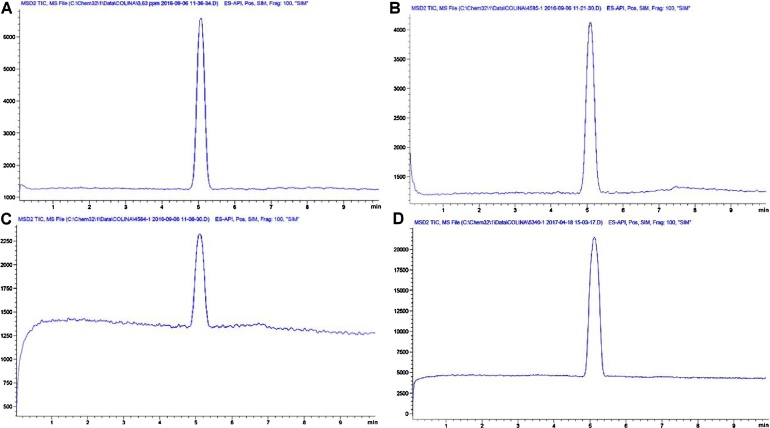
A. Choline_(*tr*_ _=_ _5.10)_ standard solution 0.63 mg L^−1^ as measured using SIM mode. Choline feed additives with values of B. (28.78 ± 1.33) g/100 g, C. (13.62 ± 0.69) g/100 g and D. (69.24 ± 3.46) g/100 g.

As ESI^+^ was employed, the counter ion is not perceived by the detection system. Later, base choline is quantified, an issue that must be taken into account during formulation. The choline base should be multiplied times 1.34 (i.e. 139.62 g mol^−1^/104.17 g mol^−1^) to obtain choline chloride. For example, quality control performed in our laboratory has found choline additives which reported far less free-choline than that recovered experimentally. Despite samples 4584 and 4585 being labeled as feed additives with 60 g choline/100 g, our obtained values ranged only from 13.6 to 28.8 g/100 g. On the other hand, a purer additive sample 5340 resulted in a concentration of 69.2 g/100 g (Fig. 2A–D). Hence, proving that our method can, in fact, be used for quality assurance. Applications of the method can be extended to total choline after applying ester hydrolysis (e.g., plant-derived feed ingredients or food for human consumption).

**Fig. 3:**
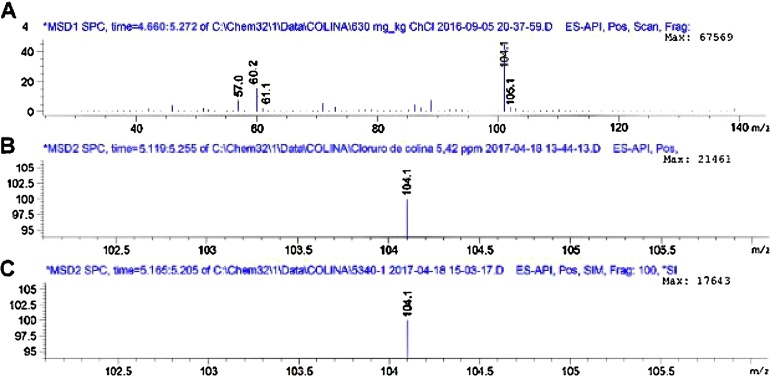
A. TIC extracted MS spectra with the common marker ions for choline [M]^+^ of 104.1 B. MS spectra SIM mode for 5.42 mg L^−1^ standard, C. MS spectra SIM mode confirming the presence of choline in sample 5340.

Quantification can be performed using molecular ion abundance (as it was the case with this report), peak area, peak height or even the daughter ion (Fig. 3A). The ion spectrum for choline is was obtained displaying the molecular ion [M]^+^ at a mass to charge ratio (*m*/*z*) of 104.1, the [M+H]^+^ ion at *m*/*z* 105.1 and the daughter ion at *m*/*z* 60.2 representing the trimethylamine cation (CH_3_)_3_NH^+^ (Fig. 3A–C). In conclusion, the proposed method exhibits great accuracy and precision and is suitable for the purpose for which it was designed.
